# Dental caries experience and associated risk indicators among Palestinian pregnant women in the Jerusalem area: a cross-sectional study

**DOI:** 10.1186/s12903-018-0628-x

**Published:** 2018-10-22

**Authors:** Elham Kateeb, Elizabeth Momany

**Affiliations:** 1 Department of Periodontology and Preventive Dentistry, Al-Quds University, Jerusalem, State of Palestine; 2 Public Policy Center, the University of Iowa, Iowa City, IA USA; 3 Department of Periodontology and Preventive Dentistry, Al-Quds University, College of Dentistry, University Main St., P.O Box 89, Jerusalem, State of Palestine

**Keywords:** Access to care, Pregnant, Dental caries, Beliefs, Attitude, Knowledge

## Abstract

**Background:**

This study described the dental caries experience of Palestinian pregnant women and examined its relationships to their oral health knowledge, beliefs, behavior, and access to dental care.

**Methods:**

Pregnant women receiving prenatal care at the Ministry of Health (MOH) centers in the Jerusalem Governorate were invited to participate in this study. Structured interviews were conducted to assess pregnant mothers’ beliefs about oral health care and their oral hygiene practices. Screening for mothers’ dental caries experience was carried out using the Decayed, Missing and Filled Teeth/Surfaces (DMFT/S) index. Univariate, bi-variate and multi-variable analysis were conducted to explain the high level of disease in this population.

**Results:**

A total of 152 pregnant women participated in this study. Mean DMFT in this sample was 15.5 ± 4.5 and an average DMFS of 31.8 ± 21. According the World Health Organization (WHO) criteria, 89% of our sample were categorized in the “Extremely High” dental caries experience. Fifty-eight percent of the DMFT scores among this sample were due to untreated dental decay, while 22% of the same DMFT scores demonstrated restorative care received by this sample. Bivariate analysis showed that mothers who completed a degree after high school had lower DMFT scores than mothers who did not (F = 4, *n* = 152, *p* = .024). In addition, mothers who believed they could lose a tooth just because they are pregnant had higher DMFT scores (*t* = − 4, n = 152, *p* = .037). The final model found that age, level of education, providers’ advice on utilizing dental care during pregnancy, and the belief that a woman can lose a tooth just because she is pregnant explained 22% of the variation in DMFT scores.

**Conclusions:**

Women in this study had a high prevalence of dental diseases and knew little about dental care during pregnancy. Faulty beliefs about dental care during pregnancy among women and health care providers were major factors in the high levels of disease.

## Background

The literature has demonstrated that women are more susceptible to dental caries during pregnancy. This finding could be due to the special conditions pregnant mothers suffer, such as increased acidity in the oral cavity, sugary dietary cravings, inadequate attention to oral health (OH) and delayed treatment [1]. In the literature, it is recorded that women who gave birth to more children show a higher percentage of ‘decays’ compared to women with only one child [2].

While pregnancy made mothers more vulnerable to OH changes, many factors independent of pregnancy may also play important roles. Three important domains presented in some conceptual models of health [3–5] include personal characteristics (e.g., demographics, socio-economic status [6]), health behaviors (e.g., health practices [7], healthcare utilization [8]), and the broader social context and environment (e.g., health care system [8]). These models suggested that dental care utilization can be a mediating factor; other factors, including demographic and personal characteristics, may influence access to care, and positive health outcomes can be impacted by easier access to professional dental care [4, 5].

In other models, some psychosocial factors were suggested, such as mothers’ stress levels (MSL) and social support (the support the mothers usually get from their families and friends) [9–11]. In one study [11], social support and MSL were both identified to be associated with OH status and OH behavior, and they were likely to influence both the decision-making process of when to seek dental care and the type of treatment to opt for. In another study [9], both social networks and MSL were identified as barriers to utilizing dental services.

Although there are many studies discussed factors related to OH of pregnant women, the complex and dynamic interactions among these factors and their influences on OH status for pregnant women are not yet fully understood. In addition, most of the previous literature did not relate those factors to pregnant women’s clinically assessed OH [12].

The importance of this study stems from the fact that there are scarce data attempted to describe the OH status, behaviors, beliefs and attitudes of pregnant women in the Levant area in general and in Palestine in particular. Hence, the present study assessed OH status among pregnant mothers attending maternal and child health care (MCHC) programs at the Ministry of Health (MOH) and examined its relationships to mothers’ OH knowledge, beliefs, behaviors, and access to dental care. These data will be helpful in planning OH prevention and intervention programs for this study population.

The current study’s specific objectives were to describe the dental caries experience among a sample of low-income pregnant women and their knowledge, beliefs and attitudes towards oral health and dental care during pregnancy. In addition, guided by our conceptual model, this study examined associations between different distal (sociodemographic and psychosocial) and proximal (oral hygiene practices and beliefs, attitudes and access to dental care) factors on the mothers’ dental caries experience.

## Methods

This study was a cross-sectional investigation employed individual in-depth interviews using a structured questionnaire among pregnant women in their 2nd and 3rd trimesters. The study was carried out by a dental public health professional team in the Jerusalem Governorate of the State of Palestine in the period from March 2015 to December 2015.

All MCHC centers at the MOH’s public clinics in the Jerusalem Governorate (*N* = 15) were included in this study. Pregnant women who attended their scheduled Obstetrician/Gynecologist (OB-GYN) appointments at the 15 clinics were invited to participate in the study. All pregnant women who were enrolled in the MCHC program in those centers were initially recruited through clinics’ listings to schedule their monthly OB-GYN appointment. Mothers who showed up on the scheduled day were asked to participate in this study. Non-responses were minimal because there was a long queue to see the OB-GYN and because mothers thought that answering some questions would help them pass the time. However, if respondents chose not to answer any of the questions, that question was excluded from the analysis.

The minimal non-response bias combined with the recruiting strategy used in this study made our final sample representative of all mothers use MCHC programs in Jerusalem governorate.

Inclusion criteria for participation included healthy women who were pregnant, in their second or third trimesters, resided in the Jerusalem Governorate and used one of the 15 MCHC centers at the MOH’s public clinics.

The study team consisted of a dental public health specialist, E.K., who conducted all the interviews in this study, and well-trained senior dental students at Al-Quds University Faculty of Dental Medicine, who conducted the clinical screening. A structured questionnaire was developed based on previous studies [13, 14] and checked for its cultural sensitivity in a sample of 13 pregnant women.

The final version of the questionnaire included questions about pregnant women’s socio-demographic data (“Age,” “Household Income,” “Level of Education,” “Employment Status,” “Insurance Coverage,” and “Number of Previous Pregnancies”), their access to dental care, their oral hygiene habits and their perception of their own OH status.

The questionnaire also included questions that assessed mothers’ beliefs about the importance of dental care during pregnancy and the influence of their OH on their own general health and on their birth outcomes. Beliefs that promoted positive OH behaviors were measured using a five-point scale (“Strongly Agree,” “Agree,” “Neither Agree nor Disagree” “Disagree,” or “Strongly Disagree”).

The questionnaire also assessed some psychosocial constructs that were measured by validated scales, such as instrumental social support and MSL [13, 14]. The MSL instrument included six items scored on a Likert scale from 1 (“Never”) to 5 (“Almost Always”). Scale’s final score of each respondent was the mean score of the ratings of the six items; thus, higher scores indicate higher levels of stress. The social support instrument comprised four items scored “Yes” or “No.” The social support instrument was calculated as a sum of the answers; each “Yes” received a “1”, and each “No” received a “0”. The higher the final result was, the more social support the mother received.

Mothers’ dental caries experience and plaque accumulation were screened using the World Health Organization (WHO) oral health survey’s community-based indices [15]. Pregnant mothers’ dental caries prevalence was assessed by the DMFT index and their dental caries experience severity by the DMFS index [15]. In addition, Russell Plaque index (PI) was used to assess oral hygiene and plaque accumulation [15]. Senior dental students who attended two calibration sessions and one hands-on training session on real patients did the OH screening for all participants. The supervisor (E.K) double checked the final recording of the OH exams.

Mothers were invited to the maternal exam room at the public clinics and were seated on a patient chair. Clinical screening followed the methods specified in the WHO pathfinder survey guidelines [15]. However, tongue depressors were used instead of periodontal probes, to exclude the need for sterilization on-site. The use of tongue depressors in field screening was validated in a previous study [16]. Plaque accumulation using PI was first evaluated, and then mothers were asked to brush their teeth; dental caries indices were examined afterward. Clinical exam data were recorded in special forms and collected with the questionnaire data at the end of each session.

Participation was voluntary, and signed paper consent forms were collected from mothers who agreed to participate in this study. Consents for participants under the age of 18 were signed by their parents/guardians. All aspects of this study, including the consent forms, were approved by the Scientific Research Ethics Committee of Al-Quds University. This study has been conducted in full accordance with the World Medical Association Declaration of Helsinki.

**Fig. 1:**
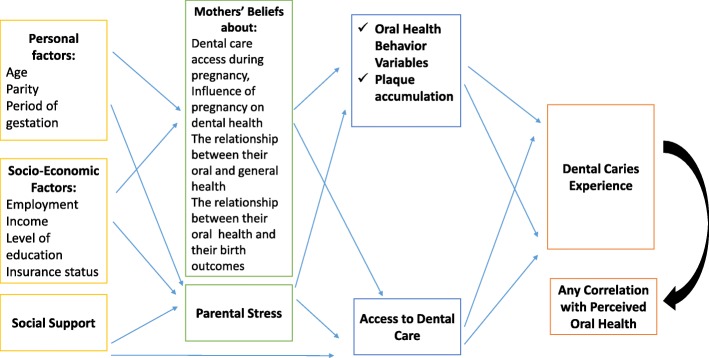
A Conceptual Model Explaining the Relationships between the Study Variables

The analysis used in this study was guided by the conceptual model shown in Fig. 1. This conceptual model was based on previous models used in the dental literature [17, 18] to explain access to dental care and OH conditions among different populations.

Independent variables included the following: social variables, demographic variables, a social support scale, employment status (“Student,” “Housewife,” “Part-time Job”, “Full-time Job”); dental insurance (“Private,” “Public,” “None”); and education (“Less than High School,” “High School,” “Two Year College,” “Four-Year College or More”). Household monthly income (less than $399, $400–$799, $800–$1199, $1200–$1599, more than $1600) was used according to the Palestinian Central Bureau of Statistics [19].

Variables describing access to dental care were as follows: 1) last dental visit within the past “6 Months,” “12 Months,” “3 Years,” “5 Years,” “Never Been to a Dentist Office,” and 2) if the mother had a dental home (a particular dentist she usually visits). Variables describing oral hygiene practices such as brushing (“Never,” “Sometimes,” “Once a Day or More”) and flossing (“Never,” “Sometimes,” “Daily”) were also used to describe oral hygiene habits (self-reported). In addition, we asked mothers to demonstrate the way they usually brush and floss their teeth to assess if they perform this task correctly or not (oral hygiene habits noted). Plaque accumulation measured by PI was treated as continuous variable; however, this variable was categorized according to WHO Oral Health Survey [15] criteria to describe mothers’ oral hygiene.

MSL and statements that described mothers’ beliefs about 1) dental care during pregnancy, 2) relationship between their oral and general health, and 3) relationship between their OH and birth outcomes were used as mediating variables, as illustrated in the conceptual model.

Dental caries experience was the dependent variable in this study, summarized by the DMFT index. DMFT index was treated as continuous data; however, for descriptive purposes, DMFT index was categorized into 4 categories according to cut points assigned by the WHO Oral Health Survey [15].

## Results

One hundred fifty-two pregnant mothers completed our in-person structured questionnaire, and 151 of these mothers did the OH clinical screening. Mothers’ ages ranged from 17 to 42 years old, with an average of 26 years (SD = 5.4). Table 1 presents socio-demographic characteristics and barriers to accessing dental care in our sample.

**Table 1:** Demographic and access to care characteristics of the study sample

Demographic characteristics	Percentages
Mother’s employment	
Working	6.6
Stay at home	86.8
Students	6.6
Mother’s education level	
Less than high school	41.1
High school diploma	25.8
Post-high school education	33.1
Monthly household income	
Less than $400	24
$400–$799	46.7
$800–$1200	19.3
More than $1200	10
Access to dental care	
Dental Insurance	
Public	4.6
Private	24.8
No insurance	70.6
Dental home	
Yes	35
No	65
Last visit to the dentist	
6 months	32.2
12 months	34.2
3 years	15.4
5 years	10.1
Never been to a dentist	8.1
Barriers to dental care (Self-reported)	
Safety concerns by family and friends	47.3
Dental costs	26.2
Time constraints	20.8
Advice by care providers not to seek treatment	32.9
Oral health is not a priority during pregnancy	25.5

The mean score of the MSL scale in this sample was 3.4 out of 5 (SD = 0.8, range = 1.2–5.00), and the mean score of the Social Support scale was 2.6 out of 4 (SD = 1.1, range = 0–4).

Oral hygiene practices and oral hygiene scores are shown in Table 2. A total of 100% of our sample had experienced dental decay. On average, participants had a DMFT score of 15.5 (SD = 5.5, range = 1–26) and a DMFS score of 31.8 (SD = 21.8, range = 1–127), with untreated decay DT of 7.9 (SD = 4.7, range = 0–20) and DS of 12.7 (SD = 10.5, range = 0–58). The FT component, which reflects the dental treatments the mother received, was 3.0 on average (SD = 3.2, range = 0–15). Dental caries experience severities, according to the WHO Oral Health Survey [15] and mothers’ perception of their own OH, are shown in Table 2.

**Table 2:** Oral hygiene practices, perceived oral health and oral health indices of the study sample

Oral hygiene practices (self-reported)	Percentages
Brushing	
At least twice a day	30
Once a day	34.6
Sometimes, irregular	32.7
Never	2.7
Flossing	
Daily	1.3
Sometimes	12
Never	86.7
Correct brushing (noted)	
Yes	73.6
No	25.7
Perceived oral health	
Poor or Fair	45.1
Average	29.1
Good or Excellent	25.8
Dental caries experience (DMFT Index)	
Very low	0.7
Low	2.0
Moderate	2.0
High	6.0
Extremely high	89.3
Oral hygiene (Plaque index)	
Good	14.6
Fair	70.1
Poor	15.3

When mothers’ perceived OH was compared to clinical measures, significant correlations were found with mothers’ dental caries experience (DMFT) (Spearman’s Rank ƿ = 0.305, *p* < 0.000).

A total of 51.8% of our sample believed that their dental problems might affect their general health. However, 75% of the same sample were not sure if a mother’s poor OH may contribute to a low-birth-weight baby or other negative birth outcomes.

In addition, although 86.8% of the mothers “Agreed ”or “Strongly Agreed” that it is important for adults to go to the dentist, even when they do not have problems with their teeth, 38% of our sample still thought it was unsafe for pregnant women to get routine dental care, such as checkups and cleanings. Moreover, 57% “Agreed ”or “Strongly Agreed” that a woman can lose a tooth just because she is pregnant.

After stratifying by socio-demographic and behavioral characteristics, disparities were found in the dental caries experience in our sample. Education was a significant factor in dental caries experience; there was a statistically significant difference between groups, as determined by one-way ANOVA (F = 4.00, *p* = 0.02). A Tukey post hoc test revealed that mothers who had a post-high school diploma had lower DMFT scores than mothers who only finished their high school diploma (*p* = 0.015). In addition, mothers’ level of education was a statistically significant factor in mothers demonstrating the correct way of brushing (Σ^2^ = 15.6, *p* = 0.048). In turn, mothers who failed to demonstrate the correct way of brushing (*t* = 2.06, *p* = 0.041) and had more plaque accumulation (*r* = .31, *p* < .0001), scored higher on the DMFT index.

As expected, older mothers and mothers who had more than one baby had higher scores on the DMFT (*r* = 0.292, *p* < 0.0001) and (*t* = 2.6, *p* = 0.01) respectively.

Mothers who had a dental home had lower DMFT than did mothers without a dental home (*t* = 2.09, *p* = 0.038). A surprising result, although it was of borderline statistical significance, was that mothers who had never been to a dentist had lower DMFT scores compared to mothers who visited a dentist in the past 6 months, based on findings from a one-way ANOVA (*F* = 2.4. *p* = 0.053; Tukey post hoc test *p* = 0.058).

When we assessed barriers to utilizing dental care during pregnancy, we found that mothers who perceived dental costs and time restrictions as important challenges had higher DMFT scores (*t* = 2.09, *p* = 0.038 and *t* = 2.11, *p* = 0.036, respectively). Mothers also reported that their general health providers’ advice about the lack of safety of visiting dentists while pregnant was another barrier to accessing dental care (*t* = 2.09, p = 0.038).

Mothers’ beliefs about OH during pregnancy were the most important factors in their high caries experience. Pregnant mothers who thought that they could lose a tooth just because they were pregnant or that it was unsafe to visit a dentist while pregnant scored higher on the DMFT index (*t* = 3.99, *p* < 0.0001). This widespread incorrect belief was also associated with more plaque accumulation (*t* = 2.372, *p* = 0.019).

Regarding psychological factors, MSL was a significant factor in increasing DMFT scores (*r* = 0.20, *p* = 0.014). Although social support and household monthly income were not directly associated with DMFT scores, they were instrumental in increasing MSL (*r* = − 2.37, *p* = 0.003 and *r* = 0.232, *p* = 0.006), respectively.

All variables that were found significant in the bivariate analysis were included in the linear regression model. Stepwise linear regression was carried out, and results were then confirmed by forward and backward regression.

After controlling all other variables, five variables explained 22% of the variation in the DMFT scores in this sample. Older mothers (β = 0.23, *p* = 0.004) who did not have a degree past their high school diploma (β = 0.17, *p* = 0.04) scored higher on DMFT index. In addition, mothers who failed to demonstrate the correct way of brushing and believed that they could lose a tooth only because they were pregnant scored higher on the DMFT index (β = 0.16, *p* = 0.039; β = 0.26, *p* = 0.001 respectively). Mothers who didn’t seek dental care during pregnancy because of their health care providers’ advice had higher DMFT scores (β = 0.16, *p* = 0.036).

## Discussion

There is enough evidence-based literature to suggest that good OH during pregnancy not only improves the quality of life of the pregnant mother but also potentially reduces complications during pregnancy and the risk of her child developing Early Childhood Caries (ECC) in the future. However, pregnant women often have misconceptions about OH during pregnancy, which prevents them from taking care of their OH or seeking professional care.

This study investigated pregnant women’s OH beliefs and behaviors and assessed their dental caries experience. The sample in this study represented mothers who used MCHC programs at the Palestinian MOH public clinics. The sample was randomly selected from the 15 centers, and mothers shared many demographic characteristics. Our results showed that most of the mothers had low levels of education, low monthly household incomes, and irregular access to dental care. As a consequence, all mothers in this sample suffered from dental caries and bad oral hygiene. Although the intensity of these oral conditions varied, the numbers demonstrated a high burden of disease.

According to the classification of the WHO Oral Health Survey Basic Methods [15], 89% of our sample were categorized in the “Extremely High” dental caries experience. Moreover, 58% of the DMFT scores among this sample were due to untreated dental decay, while 22% of the same DMFT scores demonstrated restorative care received by this sample. This finding reflects the high treatment needs in this sample; however, restorative care alone will not solve the problem. The high plaque accumulation in this sample, combined with the fact that almost 85% of the sample perceived their OH as “Poor” or “Fair”, suggests that oral hygiene education, motivation and raising awareness among pregnant women are necessary here.

Data from published literature showed significant differences in the caries experience among pregnant women in different areas of the world. The DMFT scores of pregnant mothers in some disadvantaged groups in Finland (DMFT = 18), Brazil (DMFT = 14) and Hungary (DMFT = 12.57) were very close to the DMFT scores in the current study [20–22]. In contrast, data from more representative samples of the general population in Iran (5.4) and India (3.6 and 4.8) indicated a lower burden of disease [23–25]. These vast differences in the caries experience between different areas in the world can be explained by the uniqueness of the socioeconomic and cultural structures of the samples in each study.

In Palestine, there is no data about dental caries experience among adults, except one study [26] that was conducted on a convenience sample of men and women in the commercial capital of the West Bank area in Palestine, Ramallah. Data from the previous study demonstrated lower DMFT scores in general for the same age group, 18–45 years old, with a DMFT mean of 9.03 ± 6.07. However, when subgroup analysis was carried out, women in this age group scored a DMFT mean of 8.5 ± 6.22, which is much lower than the numbers in the current study. This finding can be explained by the fact that the Ramallah study was conducted in a sample that had higher education attainment and lived in a wealthier part of the West Bank.

The high DMFT values found in this study were related to many factors in the current analysis. As documented in other literature, mothers’ level of education was detrimental to their dental caries experience; not only did mothers with post-high school education score lower on the DMFT index, but they were also able to demonstrate the correct way of brushing and had less plaque accumulation on their teeth.

The literature shows that dental care access and utilization are influenced by factors at the personal, provider, community, and organizational levels [27]. Factors such as cost and insurance status, knowledge and beliefs, perceptions of the importance of OH, and providers’ advice about dental care during pregnancy were found to be important in pregnant mothers’ access to dental care [28, 29]. Interestingly, in the current study, having a dental home was a better indicator of dental care utilization than having a recent visit in the past year. Mothers who answered “yes” to having a private dentist scored lower on the DMFT, while mothers who had a recent visit during the last 6 months or one year scored higher. This finding can be explained simply by the reason for the recent visit, being mainly due to pain, which suggests that visits to dental offices were irregular and mainly to relieve pain among mothers in this sample.

Mothers’ belief that OH is unrelated to general health or that it has no influence on their unborn children’s general health made seeking dental care not a priority in this sample, as our results demonstrate. Cost and time were the main barriers, which is expected in a sample that showed large numbers of children per family and low monthly household incomes. However, health care providers’ advice not to visit a dentist while pregnant is an unacceptable practice in light of the current understanding of the relationship between OH and general health. This finding implies that awareness needs to be extended not only to mothers but to different health care providers about the importance of OH and dental care during pregnancy.

Consistent with previous literature [30], older and multiparous mothers scored higher on the DMFT. Having more than one baby increased MSL in our sample and influenced DMFT scores directly and indirectly. In contrast, high scores on social support scale were associated with low MSL and indirectly affected DMFT in a positive way.

Mothers’ beliefs about OH during pregnancy played an important role in mothers’ OH status, especially dental caries experience. In line with previous literature [31], these beliefs were the strongest predictors of high levels of disease. The belief that pregnant women can lose a tooth just because they are pregnant was surprisingly embraced by many mothers and was one of the most solid beliefs they had about OH during pregnancy. Paradoxically, mothers also believed that visiting dentists during pregnancy for routine care is unsafe. These two beliefs and the failure to demonstrate the correct way of brushing were very detrimental factors in high disease levels.

The findings of this study demonstrated that pregnant women from disadvantaged backgrounds had the greatest burden of poor oral hygiene and dental caries experience, which is consistent with the findings in the current literature on OH status disparities in general [8, 32] and in pregnancy in particular [33].

This finding suggests that the current situation should be addressed at different levels. Educational campaigns targeting pregnant women and health care providers should be designed and incorporated in pre- and postnatal programs to promote the importance and safety of dental care during pregnancy. These interventions are necessary but insufficient; multilevel national programs that address the social, economic and organizational factors that influence OH status provide other important venues to alleviate this problem from its root factors.

The strengths of this study were random sampling and combining clinical screening with self-reports to assess OH status. Although we are confident of the generalizability of our results to the population of pregnant women in the Jerusalem Governorate, we cannot extrapolate beyond this geographical area. Additionally, the small sample size may underestimate the influence of some significant variables in the final analysis.

Another limitation was that oral hygiene practices were self-reported, which made them susceptible to social desirability. Moreover, methods used in this study had some limitations; periodontal conditions were only assessed by the GI index, which measures gingivitis but not periodontitis. In addition, tongue depressors were used to assess dental caries among pregnant women instead of periodontal probes. This method has high specificity but low sensitivity [16].

## Conclusions

The results from this study provide evidence that emphasizes the importance of OH promotion and disease prevention programs during pregnancy. High levels of dental caries experience and poor oral hygiene practices in this sample justify the need to incorporate OH education and motivation interventions in the pre- and postnatal care programs administered by the MOH in public clinics. Findings regarding the factors that explain the high burden of disease, such as faulty beliefs, incorrect practices, access to dental care and other health care providers’ perspectives on dental care during pregnancy, can be used to tailor OH programs that benefit pregnant mothers the most.

## Abbreviations

DMFS: Decayed, Missing, and Filled Surfaces
DMFT: Decayed, Missing and Filled Teeth
ECC: Early Childhood Caries
MCHC: Maternal and Child Health Care
MOH: Ministry of Health (MOH)
MSL: Mothers’ Stress Levels
OH: Oral Health
PI: Russell's Plaque index
WHO: World Health Organization
